# On Modern Progress in Ophthalmic Medicine and Surgery

**Published:** 1894-09-08

**Authors:** Robert Brudenell Carter

**Affiliations:** Consulting Ophthalmic Surgeon to St. George's Hospital


### ON MODERN PROGRESS IN OPHTHALMIC MEDICINE AND SURGERY.

By ROBERT BRUDENELL CARTER, F.R.C.S., Consulting Ophthalmic Surgeon to St. George's Hospital.

LAMINAR CATARACT—(*Continued*).

WHEN laminar cataract covers too large an area of the lens to admit of sufficient improvement of vision by the formation of a lateral pupil, the removal of the lens itself is the only surgical procedure which remains for consideration, and this, as mentioned in the last paper, may be accomplished either by absorption or by suction. In order to obtain absorption, all that is required is to puncture the anterior capsule in such a manner as to expose the lens tissue to the action of the aqueous humour, by which it will be gradually dissolved; and the chief precaution necessary is so to regulate the size of the puncture that the eye may not be oppressed by the presence, in the anterior chamber, of sufficient lens material to become a source of irritation or inflammation. The pupil being first fully dilated, the surgeon introduces a very fine needle through the cornea, on the horizontal meridian, at a point about a line distant from the temporal margin, and directs its point to the centre of the lens, where he makes a single linear incision through the anterior capsule. The needle is at once withdrawn, and the eye closed by any suitable dressing. At the end of twenty-four hours the needle puncture in the cornea will be closed, while that in the anterior capsule will be visible as a white streak, through which a minute portion of opaque lens matter will be projecting into the anterior chamber. The pupil must be kept fully dilated by the daily use of atropine, and the projecting lens matter will presently be absorbed, and replaced by the protrusion of more. In some cases, the single puncture may be sufficient, and the whole of the lens may gradually disappear; but in others the wound in the capsule will become closed, and the process of absorption will be arrested. The puncture should then be repeated, and the needle may generally be used with greater freedom on the second occasion than on the first. Ultimately, in favourable cases, the whole of the lens will disappear, and the pupil will be occupied by a film of capsule, more or less opaque. This may then be torn through by two needles, as is done with the films which are sometimes left after the extraction of senile cataract, and the patient, given the aid of artificial lenses, may be expected to have excellent sight.

The operation of absorption, when performed upon children or young persons, is often followed by the most admirable results, but it is open to the objection of requiring a long time, possibly three months or more for each eye, during the whole of which period the eye is irritated by the presence of lens matter in the anterior chamber, and is liable to attacks of inflammation, which may commence as iritis, and may extend backwards to the choroid, and ultimately destroy the sight. Human nature being what it is, the parents or friends of the patient, who may usually be trusted to watch over his or her condition for a week or two, are apt after a time to relax their vigilance, and then, unless the surgeon is able to see the case constantly, irreparable mischief may ensue. The risks incidental to absorption are therefore not inconsiderable, and the operation should only be performed in circumstances which permit of a close and somewhat prolonged medical attendance, coupled with strict domestic supervision. It was on account of these disadvantages, and in order to bring the time required for removal of the lens within narrower limits, that Mr. Pridgin Teale introduced into this country the operation of suction, which has been practised from time immemorial in Persia, and which he modified in such a way as to bring it into harmony with the requirements of modern surgery.

The principle underlying suction is that the lens is in the first place completely broken up by needles, and is then left for a few days to the action of the aqueous humour, by which it becomes converted into a pulp. This pulp, instead of being left for absorption, is removed from the eye by a suction tube, which may be actuated either by a syringe, or by the mouth of the operator, the latter plan being by far the best in the majority of cases.

In order to perform suction, the pupil should be fully dilated, and two needles should be introduced through the cornea, one near each extremity of the horizontal meridian. The needle introduced on the temporal side is applied to the nasal half of the lens, and that introduced on the nasal side is applied to the temporal half of the lens. Each needle is made to divide its portion of the lens by a series of closely set parallel cuts or strokes, which are carried as deeply as possible, but not so deeply as to go through the posterior capsule into the vitreous body. This part of the procedure is one of considerable delicacy, because, if the needles go too deep, and permit the vitreous body to mix with the broken lens, the softening action of the aqueous humour will be seriously retarded; while, if the needles do not go deep enough, the posterior layers of the lens will escape division, and will not become so completely softened as is desirable. Nothing but practice, guided by an adequate knowledge of the anatomy of the structures concerned, will enable the surgeon to do just enough, and to avoid doing too much.

The lens being completely cut to pieces, the eye may be closed for twenty-four hours, at the end of which time the anterior chamber will be filled, and the margins of the pupil commonly concealed, by a flocculent looking, opaque, pultaceous mass, which consists of the softened and swollen fragments of the lens. Atropine must be applied, and the eye watched carefully for a few days. If there should be pain or redness, suction should be performed without delay, but in the absence of any signs of irritation or inflammation it is better to wait, in order to secure such a degree of softening of the fragments as will allow them to pass easily through the suction tube. Whenever possible, I am accustomed to wait for a week, but have often been compelled to complete the operation at an earlier period.

The suction instrument is a small silver tube, slightly curved, about an inch long, semi-circular in section, terminating in a smooth and rounded closed extremity, and having an oval aperture near this extremity on its upper or flat surface. At the other end it is expanded into a conical shoulder, by which it is cemented upon a glass tube six inches long, and as large as a cedar pencil, which serves as a handle, and to which an india-rubber tube, terminating in a mouthpiece, is attached. Two small lateral notches are cut in the silver, continuous with the minor axis of the oval opening, so that the latter cannot be closed by contact with the posterior surface of the cornea.

